# First record of a termite–fungus association in Oligocene evergreen oak wood type (*Quercoxylon*) from Central Europe

**DOI:** 10.1093/aob/mcag141

**Published:** 2026-05-26

**Authors:** David Krejčíř, Jakub Sakala, Vladimír Gryc, František Zelenka, Josef Kašák

**Affiliations:** Department of Geological Sciences, Faculty of Science, Masaryk University, Kotlářská 2, 611 37, Brno, Czech Republic; Institute of Geology and Paleontology, Faculty of Science, Charles University, Albertov 6, 128 43, Prague 2, Czech Republic; Department of Wood Science, Faculty of Forestry and Wood Technology, Mendel University, Zemědělská 3, 613 00, Brno, Czech Republic; VSB – Technical University of Ostrava, 17. Listopadu 2172/15, 708 00, Ostrava, Czech Republic; Department of Forest Protection and Wildlife Management, Faculty of Forestry and Wood Technology, Mendel University, Zemědělská 3, 613 00, Brno, Czech Republic

**Keywords:** Bioindication, Fagaceae, Isoptera, fossil wood, coprolites, heartwood rot, Palaeogene

## Abstract

**Background and Aims:**

The Eocene–Oligocene transition (∼33.5 Ma) brought major climatic change with lasting effects on terrestrial ecosystems. Termites are important indicators of palaeoenvironmental conditions in terrestrial ecosystems, and here we report hexagonal termite coprolites (*Microcarpolithes hexagonalis* Vangerow) preserved within anatomically well-characterized silicified evergreen oak wood type (*Quercoxylon* aff*. helladae*) from the Oligocene Menilite Formation of Central Europe.

**Key Results:**

The material, recovered from Quaternary terraces of the Bečva River (Czech Republic), represents redeposited silicified wood originally derived from Menilite facies. Thin sections and micro-computed tomography reveal termite galleries filled with coprolites, closely associated with clamp-bearing hyphae and pocket-rot features consistent with white-rot Basidiomycota, documenting a close spatial co-occurrence of termite-produced coprolites and fungal structures within a single specimen and providing new insight into decay processes in Oligocene forest ecosystems.

**Conclusions:**

The observed association highlights the complexity of saproxylic communities in Oligocene forests of Central Europe and is consistent with the presence of locally warm, humid (subtropical) conditions despite global cooling.

## INTRODUCTION

The Eocene–Oligocene transition (∼33.5 Ma) marked the shift from greenhouse to icehouse conditions driven by Antarctic glaciation and global cooling (Eldrett *et al*., 2007, 2009; Zachos *et al*., 2008). This ∼0.5-Myr interval caused profound environmental reorganization in Central Europe, with stronger seasonality, regional aridification and restructuring of terrestrial ecosystems (Pound *et al*., 2012). While both marine and continental records document progressive cooling and drying, sclerochronological δ^18^O profiles from *Glycymeris* shells reveal pronounced seasonality and episodic humid phases during the Rupelian (Walliser *et al*., 2015). Plant macrofossils likewise indicate that humid–subtropical vegetation persisted locally in parts of Central Europe throughout the early Oligocene (Kovar-Eder, 2003; Teodoridis and Kvaček, 2015).

Kalotermitid termites are saproxylic, i.e. dependent on dead wood at various stages of decay, and play an important role in decomposition processes and nutrient cycling, with their success relying on complex symbioses with gut microorganisms and fungi that enable efficient lignocellulose degradation (Roisin, 2000; Wilson and Hölldobler, 2005; Rouland-Lefèvre *et al*., 2006; Engel, 2019). Because their ecology is tightly bounded to tropical and subtropical environments, termite ichnofossils – particularly coprolite-filled galleries in wood – serve as valuable palaeoenvironmental indicators. Their presence in Palaeogene deposits demonstrates that warm subtropical conditions persisted in Central Europe even during intervals of global cooling (Genise, 2017; Zhao *et al*., 2020). In the fossil record, termites are represented not only by ichnofossils but also as body fossils in fine-grained lacustrine deposits (Jarzembowski, 1981; Martínez-Delclòs and Martinell, 1995; Engel *et al*., 2007) and amber inclusions (Engel *et al*., 2011; Engel *et al.*, 2016; Zhao *et al*., 2020, 2021). Termite nests (Genise and Bown, 1994; Duringer *et al*., 2007; Kaulfuss *et al*., 2010; Roberts *et al*., 2016) and coprolite-filled galleries (Jarzembowski, 1990; Rozefelds and Baar, 1991; Sutherland, 2003; Francis and Harland, 2006; Pires and Sommer, 2009; Colin *et al*., 2011; Massini and Pujana, 2013; Jud *et al*., 2017; Moreau *et al*., 2020) occur from the Cretaceous to the Pliocene, documenting the long-term persistence of termite activity across diverse ecosystems and forming part of the broader evolutionary history of burrowing invertebrates (Humphreys, 2003).

Hexagonal termite coprolites, owing to their distinctive morphology and unequivocal biological origin, represent excellent palaeoenvironmental indicators with a fossil record spanning multiple intervals and regions. The oldest reliable occurrences date back to the Early Cretaceous (Pires and Sommer, 2009; Heřmanová *et al*., 2021). Mesozoic records, particularly from the Cretaceous, include well-documented examples from the Late Cretaceous (Campanian–Maastrichtian) of Texas (Rohr *et al*., 1986), the Albian–Cenomanian of Australia (McLoughlin *et al*., 2024), the Lower Cretaceous (Hauterivian–Aptian) Upper Strzelecki Group of southeastern Australia (Edwards *et al*., 2025), the Lower Cretaceous (Aptian) Huolinhe Formation of eastern Inner Mongolia, China (Dong *et al*., 2022), *Paraphyllanthoxylon*-type woods from British Columbia (Conician) (Jud *et al*., 2017), silicified conifer woods of the Kachaike Formation (Albian) in Argentinean Patagonia (Greppi *et al*., 2023) and Early Cretacous (Barremian) softwoods of the UK (Francis and Harland, 2006). Cenozoic (Palaeogene) occurrences are known from the Late Cretaceous–Palaeogene (Cenomanian) Klikov Formation of the Czech Republic (Heřmanová *et al*., 2021), Eocene–Oligocene (Priabonian–Rupelian) conglomerates of southern France (Moreau *et al*., 2020) and Oligocene (Rupelian) deposits of Australia (Rozefelds and Baar, 1991). Neogene occurrences include Miocene strata of Argentina (Massini and Pujana, 2013), fossil woods (Aquitanian–Burdigalian) from New Zealand (Lindquist and Isaac, 1991; Sutherland, 2003) and Brazilian fossil woods (Pires and Sommer, 2009), whereas Pliocene opalized woods from California were described by Rogers (1938).

Collectively, these records highlight the long evolutionary continuity of this distinctive trace fossil and provide a framework for interpreting new Oligocene finds from Central Europe.

Basidiomycota are among the most diverse and widespread fungal lineages and play a major role in decomposition and nutrient cycling in terrestrial ecosystems (Lindahl and Tunlid, 2015). Fungal decay in fossil wood is known throughout geological history, with preservation from hyphae and spores to reproductive structures. The oldest record comes from *Callixylon* in the Upper Devonian (Stubblefield *et al*., 1985) and Permo-Triassic gymnosperms from Antarctica (Stubblefield and Taylor, 1986). Jurassic examples include tyloses with fungal interactions in a conifer from Antarctica (Harper *et al*., 2012), *Xenoxylon* from the USA (Xie *et al*., 2023), and diverse decay morphologies in Jurassic–Cretaceous woods from Asia and South America (Boura *et al*., 2013; Feng *et al*., 2015; Wan *et al*., 2016*a*, *b*; Rombola *et al*., 2022). Later records include Palaeocene Araucariaceae (Pujana *et al*., 2015), Eocene ascomycetes (Wilkinson, 2003), the Oligocene *Tetracoccosporium eocenum* (Biradar and Mahabale, 1972), and Miocene–Pliocene wood-decaying fungi from New Zealand, India and Italy (Lindquist and Isaac, 1991; Sen, 2020; Greppi *et al*., 2022). Collectively, these records emphasize the persistence and ecological significance of wood-decaying fungi through geological time.

Although termite damage and fungal decay are widespread in fossil woods (Genise, 2017), no previous study has documented hexagonal termite coprolites directly associated with fungal structures in well-preserved Palaeogene wood from Central Europe. Here we report the first such occurrence, providing direct evidence of a termite–fungus association and new insight into decay processes within Oligocene forest ecosystems. Evergreen Fagaceae, including *Quercoxylon* and *Eotrigonobalanus*, were widespread across Europe and extended into parts of Asia during the Palaeogene, but today persist mainly as relict taxa in subtropical and tropical regions, while also occurring in warm-temperate ecosystems, including Mediterranean-type plant communities such as Californian chaparral and oak woodlands (Velitzelos *et al*., 1999; Napton, 2012; Nixon, 2006; Sari *et al*., 2021). This study details the first such occurrence from Central Europe, offering direct evidence of a termite–fungus association and demonstrating the methodological potential of silicified wood, using combined anatomical analysis, thin section microscopy, and micro-computed tomography (micro-CT), to document interactions that are otherwise rarely preserved in the fossil record.

## GEOLOGICAL SETTING

**Fig. 1.**
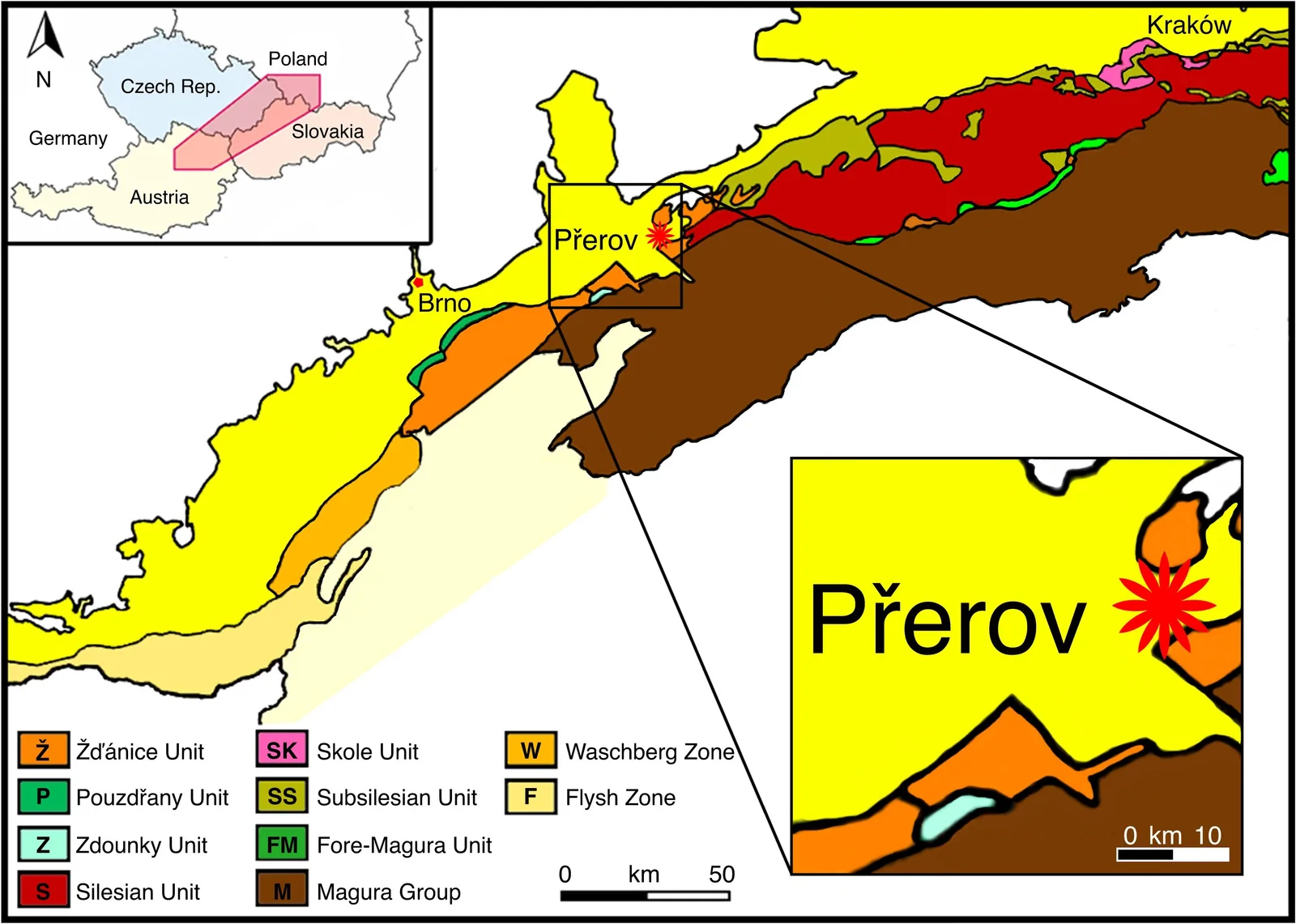
Geological map of the Western Carpathians showing the position of the Přerov fossil locality (star symbol) near the boundary between the Žďánice Unit and the Carpathian Foredeep. Modified from Jirman *et al.* (2020).

The fossil site near Želatovice (Přerov County, northern Moravia; 49°27′06.6″N, 17°27′28.7″E; Fig. 1) lies within the Outer Flysch Belt of the Western Carpathians (Pícha and Stráník, 1999; Pícha *et al*., 2006). Although collected from Quaternary terraces of the Bečva River, the wood shows strong silicification and a bituminous odour, indicating redeposition from the Oligocene Menilite Formation, which is widespread in Moravia and yields both marine and terrestrial fossils. Anatomically comparable silicified woods have been documented from Loučka (Jirman *et al*., 2020), Krumvíř, Lipník nad Bečvou (Bubík *et al*., 2019), Hranice, Měnín, Žabčice, Mouchnice and other nearby localities (Továrková, 2011), consistently pointing to a Menilite source. Palaeobotanical evidence is congruent: the Šitbořice Beds with redeposited Menilite sediments yielded leaves of *Eotrigonobalanus furcinervis*, the foliar analogue of *Quercoxylon helladae* (Stráník *et al*., 1974; Kvaček and Bubík, 1990). In contrast, Miocene oaks from Moravia are only weakly silicified, show distinct growth rings and represent deciduous taxa derived from Badenian tuffs (Továrková, 2009, 2011).

### Characteristics of the Menilite Formation

The Menilite Formation was deposited during the Early Oligocene in isolated Paratethyan basins under dysoxic to anoxic conditions with restricted circulation (Roth, 1981; Golonka *et al*., 2006). Sedimentation occurred mainly at mesopelagic depths (200–1000 m), as indicated by fish taxa with photophores (Brzobohatý, 1981). The succession is dominated by organic-rich, commonly silicified pelites, with silica derived from diatoms and sponge spicules. Lithologies include laminated claystones, siliceous shales, marls and tuffs, whereas carbonates are scarce. Regionally, the Menilite Formation extends across the Žďánice, Silesian and Subsilesian nappes and into Slovakia, Hungary, Romania, Ukraine and southeastern Poland, typically reaching thicknesses of <100 m (Krhovský, 1981; Krhovský and Djurasinović, 1993; Stráník, 1981).

### Sedimentary and palaeobotanical context

The Menilite Formation comprises alternating bituminous shales, siliceous claystones, and marl-rich beds, reflecting fluctuating redox conditions and variable biogenic productivity in deep-water, dysoxic to anoxic settings (Švábenická *et al*., 2007). Episodic blooms of siliceous plankton – radiolarians, diatoms and sponges – provided silica that promoted selective silicification of both marine and terrestrial material (Chlupáč *et al*., 2002). Rapid burial under limited bioturbation further facilitated the exceptional permineralization of plant remains described here.

The flora of the Šitbořice Member comprises numerous angiosperms, notably *Eotrigonobalanus* (*Dryophyllum*) *furcinervis* (Rossm.) Walther & Kvaček, *Platanus neptuni* (Ettingsh.) Bůžek, Holý & Kvaček, *Daphnogene cinnamomifolia* (Brongniart) Unger, *Laurophyllum pseudoprinceps* Weyland & Kilpper and *Engelhardia* (*Palaeocarya*) *orsbergensis* (Weber) Jähnichen, Mai & Walther, accompanied by *Quercus* sp. and *Apocynospermum striatum* Reid & Chandler. Conifers include *Taxodium* cf. *balticum*, *Sequoia abietina* (Brongniart) Knobloch and *Pinus* sp. (Kvaček and Bubík, 1990).

## MATERIALS AND METHODS

### Sample preparation and microscopy

We processed 92 fossil wood specimens, cut with a diamond saw, ground (F80–F1200) and polished with cerium oxide. Thin sections of three representative samples (Pr_17, Pr_42, Zab_183 reference) were prepared in transverse (TS), radial (RLS) and tangential (TLS) planes (50–150 µm). Polished fragments were studied with a Nikon SMZ-2B stereomicroscope and thin sections with a Nikon Eclipse E400 light microscope. Images were obtained using an EPSON V600 scanner and a Leica DM2000 microscope with a DFC295 camera.

**Fig. 2.**
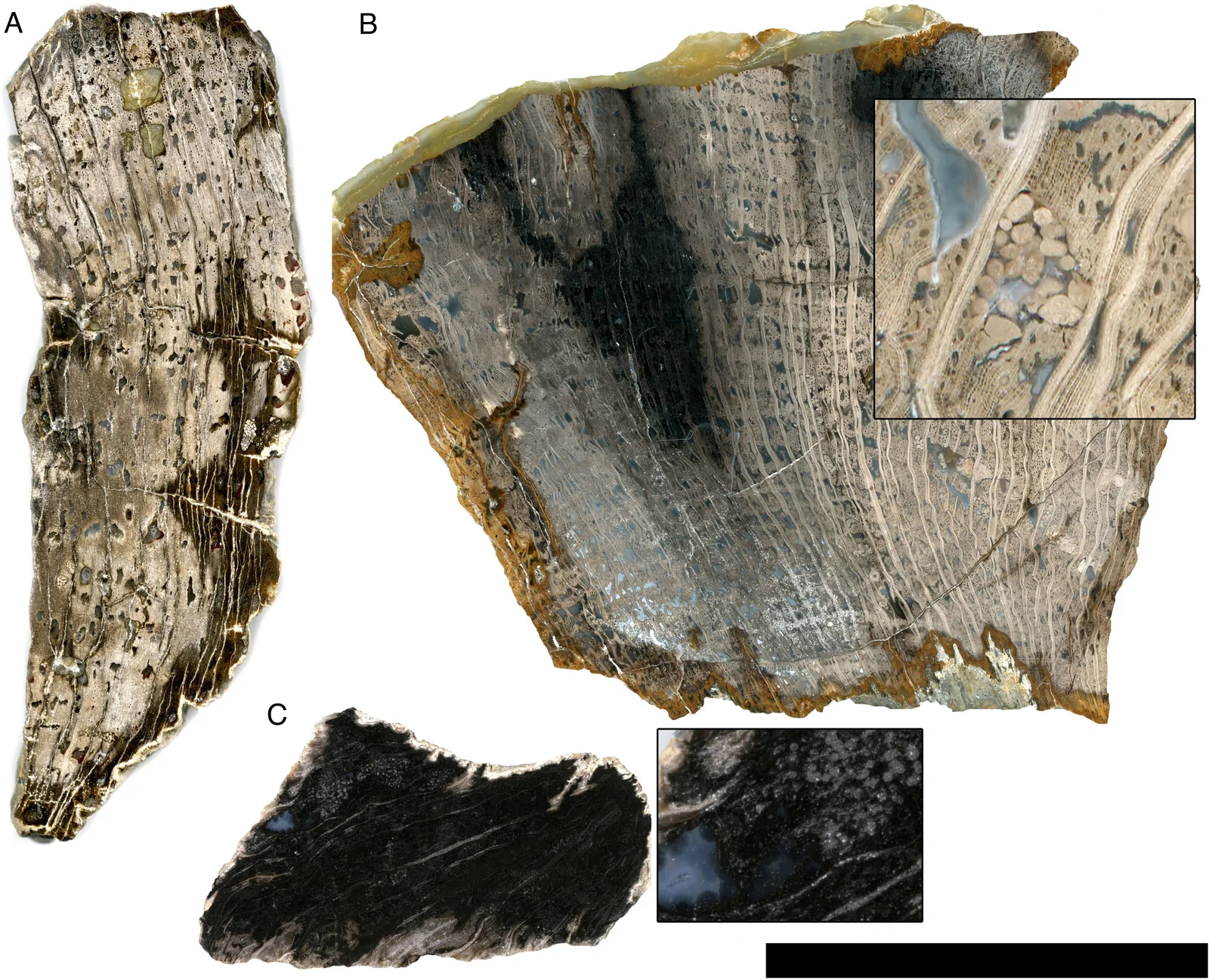
Polished transverse sections of fossil *Quercoxylon* aff. *helladae* from Moravia. (A) Pr_17, Přerov (Martin Charamza); (B) Zab_183, Žabčice (reference sample); (C) Pr_42, Přerov (Martin Charamza). Scale bars = 4 cm.

Two anatomically similar wood samples with insect coprolites and fungal remains were selected for detailed study. Sample Pr_17 (Fig. 2A), lighter and more translucent, was better suited for coprolite morphology, whereas Pr_42 (Fig. 2C), darker and more heavily colonized, preserved extensive fungal hyphae. Both originate from the Přerov locality, share consistent anatomical traits, and were cross-validated to account for intraspecific variability and preservation.

### Anatomical and taxonomic analysis

Wood anatomy was measured in ImageJ following International Association of Wood Anatomists IAWA committee,^1989^ and compared with both fossil and extant references (e.g. Cutler, 1964; Iamandei *et al*., 2012; Wheeler and Manchester, 2021). For comparison, 39 thin sections of *Quercoxylon* aff. *helladae* Petrescu, Velitzelos & Stavropodis from the Hamburg University collections were examined. Primary anatomical classification and subsequent taxonomic assignment were based on tangential sections, which allowed a detailed assessment of ray structure. Additional comparisons were made with recent thin sections of *Trigonobalanus excelsa* Lozano, Hern. Cam. & Henao and *T. verticillata* Forman.

### Coprolite and micro-CT analysis

A total of 67 termite coprolites were examined in cross-section and compared with fossil and extant analogues (Rogers, 1938; Massini and Pujana, 2013; Jud *et al*., 2017; Greppi *et al*., 2022). Selected samples (6.14 × 5.07 × 15.25 mm) were scanned by micro-CT (Heliscan), enabling 3D reconstructions of galleries and volumetric analysis of 23 coprolites. For comparison, a still image was extracted from a publicly available video of *Kalotermes flavicollis* (Fabricius) colony behaviour (Tretyakov, 2020).

### Fungal identification

Fungal structures were identified by comparative morphology with reference to published descriptions of fossil and extant wood-decaying fungi (e.g. Stubblefield and Taylor, 1986; Lindquist and Isaac, 1991; Srivastava, 2008; Feng *et al*., 2015; Biswas *et al*., 2020; Philippe *et al*., 2022).

Provenance and taxonomic attribution were constrained by (1) the distribution of the Žďánice Unit and its reworked deposits, (2) the proximity of samples to Menilite outcrops and (3) corroborating palaeobotanical evidence, notably leaf impressions of the same taxa within the Žďánice Unit.

## RESULTS

Ninety fossil wood specimens were collected from an ∼40 × 60-m area of Quaternary terrace deposits near Přerov. The assemblage exhibits consistent taphonomic features: variable silicification with chalcedony- or quartz-filled cavities, grey–brown patina where abrasion is minimal, and polished section colours ranging from white and brown to dark blue and black. Evidence of biological degradation – including insect borings, fungal galleries and fractures – occurs in 33 specimens (Appendix 1).

Of the 90 specimens, 52 were gymnosperms (58 %), 31 angiosperms (34 %), two *Palmoxylon* (2 %), one *Osmunda* (1 %) and four unassigned (4 %). Among the angiosperms, 17 specimens were assigned to *Quercoxylon* and five to *Platanoxylon*; six of the *Quercoxylon* specimens contained coprolites hexagonal in cross-section. The identified taxa comprise Cupressaceae and Pinaceae among gymnosperms, and *Platanoxylon* and Fabaceae-type wood among angiosperms. The distribution, stratigraphic assignment, and taxonomic composition of fossil wood localities in Moravia are summarized in Table 1.

**Table 1.** Fossil wood localities in Moravia (Appendix 2) with stratigraphic assignment, taxonomic affiliation and depositional context.

Stratigraphic assignment	Angio	Conif	Both	Oli	Mio	Both	*In situ*	Sec.	Žďán. un. (km) direct.
Přerov			X	X				X	Y 6 E
Pavlovice u Přerova		X		X			X		Y 0
Lipník N. Bečvou		X		X				X	Y 6 SE
Hranice		X		X				X	Y 2 E
Loučka		X		X			X		Y 8 SE
Soběchleby		X		X			X		Y 0
Kelč		X	?	X			X		Y 0
Kroměříž-Zdounky			X	X				X	Y 2 W
Vážany N. Litavou	X			X				X	Y 1 S
Měnín			X			X		X	Y 5 E
Žabčice			X			X		X	Y 4.5 E
Velké Němčice-Šitbořice				X			X	X	Y 0
Krumvíř		X		X			X		Y 0
Nemotice-Mouchnice			X	X				X	Y 1 W
Mikulov			X	X				X	Y 0

‘*In situ*’ = preserved within Menilite strata (not necessarily in growth position); other occurrences = reworked. ‘Žďán. un.’ = Žďánice Unit nearby. The last two columns indicate presence of the Žďánice Unit and distance/direction to the nearest Menilite outcrop (‘0’ = within or adjacent; data from geological map, 1:50 000 (Czech Geological Survey (Česká geologická služba), 2025). Abbreviations: Angio = angiosperm; Conif = conifer; Both = angiosperm + conifer; Oli = Oligocene; Mio = Miocene; Sec. = secondary occurrence (reworked); Y = yes, presence confirmed; X = presence in record (literature or observation); ? = uncertain; Directions (E, S, W, N) = east, south, west, north.

### Fossil wood

*Family:* Fagaceae Dumortier, 1829

*Genus: Quercoxylon* R. Kräusel, 1939

*Species: Quercoxylon (Lithocarpoxylon)* aff. *helladae Petrescu, Velitzelos & Stavropodis*

*Materials*

Two fossil wood specimens, designated Pr_17 and Pr_42 (Fig. 2), are housed in the collections of the Department of Geological Sciences, Faculty of Science, Masaryk University, Brno (Czech Republic).

*Macroscopic description*

Samples Pr_17 (12 × 4.5 × 8 cm) and Pr_42 (5 × 3 × 5 cm) both contain coprolite-filled galleries and fungal remains. Surfaces bear a white to yellow patina, while polished sections range from light to dark brown with grey to black patches.

*Microscopic description*

*Sample Pr_17* (Figs 2A). Growth rings are indistinct. Smaller vessels occur locally and only rarely; however, their frequency and distribution cannot be reliably assessed due to pronounced pathological alteration of the wood. Wood diffuse–porous, occasionally forming flame-like vessel arrangement. Vessels oval, rounded or elliptical in cross-section, mainly solitary. Vessel walls 5–10 µm thick. Vessel lumina 66–190 µm (*n* = 46, mean=134 µm, s.d. = 27 µm) in tangential diameter and 76–255 µm (*n* = 33, mean=185 µm, s.d. = 37 µm) in radial diameter. Mean vessel frequency 3.6 vessels mm^–2^. Vascular cell length 144–869 µm. Perforation plates: exclusively simple. Intervessel pits bordered, circular, alternate, quite dense, small to medium, 3.4–8 µm. Vessel-ray pits with much reduced borders to apparently simple: pits horizontal (scalariform, gash-like) to vertical (palisade). No helical thickenings present. Tyloses in vessels frequent. Fibres thin- to thick-walled, non-septate. Vasicentric tracheids present. Axial parenchyma abundant, banded reticulate (mostly uniseriate bands of parenchyma regularly spaced), apotracheal diffuse-in-aggregates, scanty paratracheal. Prismatic crystals in chambered axial parenchyma present.

**Fig. 3.**
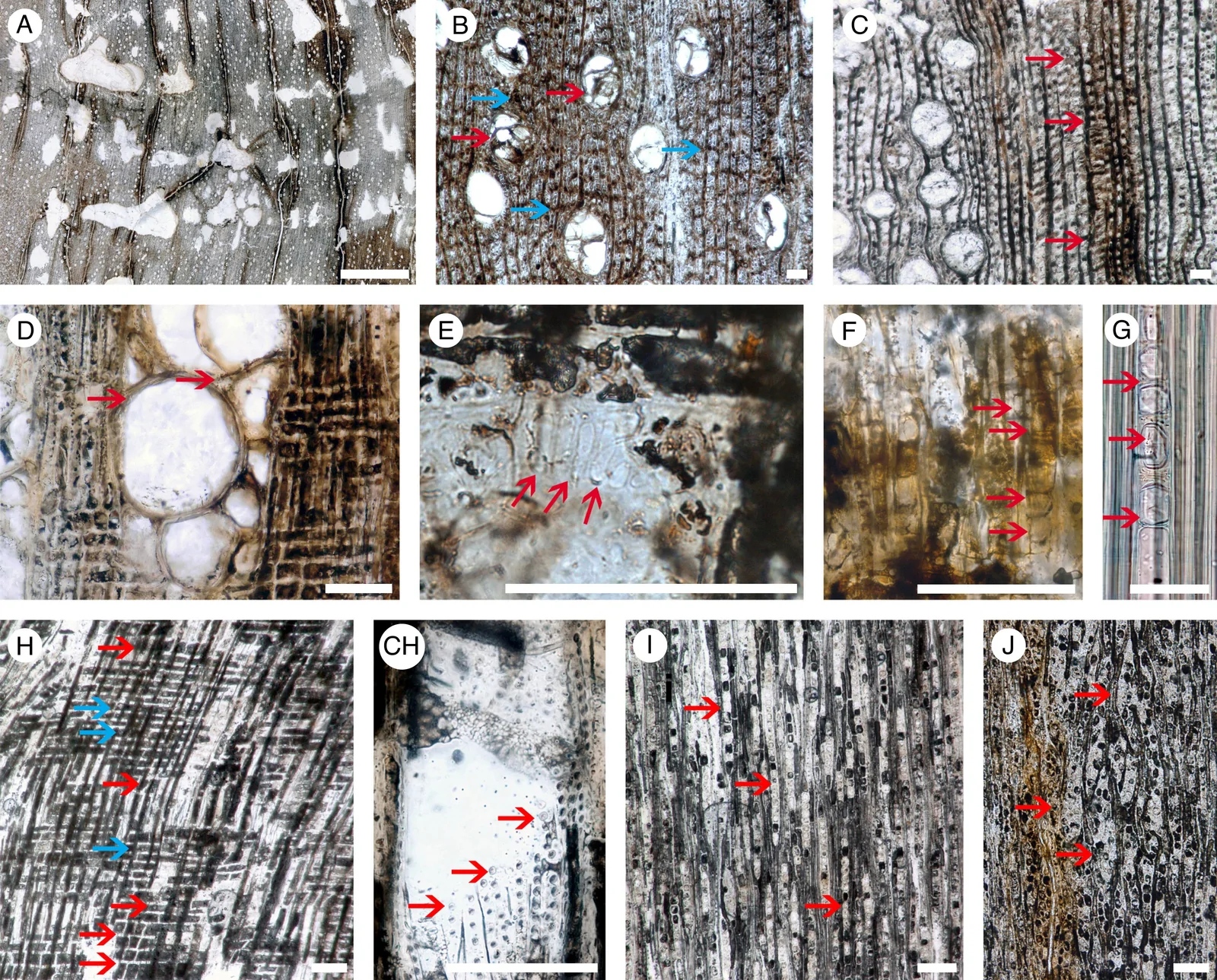
Pr_17. Wood anatomy of *Quercoxylon* aff. *helladae*. (A–C) Transverse sections; (D, E, H) radial sections; (F, G, CH, I–J) tangential sections. (A) Diffuse-porous wood with vessels in radial to flame-like patterns; growth rings absent. (B) Reticulate axial parenchyma (arrows) and tyloses (arrow). (C) Aggregate ray. (D) Tyloses in vessel lumina (arrow). (E) Palisade-type vessel-ray pits. (F) Crystals in axial parenchyma. (G) Crystal in axial parenchyma of modern *Trigonobalanus excelsa* for comparison. (H) Heterocellular ray with upright, square and procumbent cells. (CH) Vessel lumen encircled by vasicentric tracheids (arrows). (I) Aggregate rays mostly uniseriate, rarely biseriate, with transition to semi-compound rays. (J) Semi-compound rays associated with aggregated vessels. Scale bars: A = 2 cm; B–F, H–J = 100 μm; G = 50 μm.

Rays are heterocellular, with body ray cells predominantly procumbent and a single row of upright marginal cells. Two principal ray types are present: uniseriate rays and multiseriate rays. Vessel-associated rays are semi-compound, typically 2–4-seriate, occasionally reaching 5–6-seriate, and may form short vertical aggregates of semi-compound rays. Ray width ranges from 23 to 42 µm (*N* = 44, mean = 32 µm, s.d. = 4.6 µm), and ray height varies between 192 and 630 µm (*N* = 49, mean = 375 µm, s.d. = 91 µm), comprising 7–27 cells in height. Ray frequency is 16–21 rays mm^–1^ in the uniseriate portion and 27–28 rays mm^–1^ in aggregate zones. Ray cell dimensions (height × width × length) are as follows: square cells 21–31 × 16–46 × 41–94 µm; upright cells 28–68 × 27–56 × 40–76 µm (Fig. 3).

*IAWA features:* 2 5 7 9 13 22 25 32 42 46 50 56 60 66 69 77 78 87 96 97 101 102 302 106 116 142

*Comparison with fossil woods*

A combination of anatomical features, including diffuse porosity, indistinct or absent growth rings, simple perforation plates, alternate intervessel pits, vessel-ray pits with reduced borders, absence of helical thickenings, vasicentric tracheids, diffuse-in-aggregates axial parenchyma and semi-compound to aggregated rays, is characteristic of the fossil genera *Lithocarpoxylon* Petrescu and *Quercoxylon* Hofmann (Fagaceae; InsideWood, 2004–onwards). Fossil oak taxa used for comparison were selected based on growth-ring distinctness, vessel density, porosity type, cellular composition of vessel ray, occurrence of crystals in axial parenchyma and geological age.

A key diagnostic criterion for distinguishing fossil oaks concerns the architecture and cellular composition of vessel rays (Mantzouka *et al*., 2021). Evergreen members of *Trigonobalanus* – including the fossil genera *Quercoxylon* and *Lithocarpoxylon* – are characterized by semi-compound and aggregated rays (Wheeler *et al*., 2022) that result from the partial vertical fusion of uniseriate rays, forming broader heterocellular structures typically 2–10 cells wide. Table 2 summarizes the key diagnostic characters discussed here, whereas a comprehensive comparison of all examined fossil and extant oak woods is provided in Table A1.

**Table 2.** Diagnostic wood anatomical features used for comparison.

	Por	IVP	AP	Ex1s	Agg	Ray2	RayCmp	RT	Cry	Age
**Fossil**										
Pr_17	D	A	DA, B, ScP	Y	Y	N	He	S-C, C-A?	AP	Oli
Pr_42	D	A	DA, B, ScP	Y	Y	Y	He	S-C, C-A	AP	Oli
Zab_183	D	A	DA, B, ScP	Y	Y	Y	He	S-C, C-A	AP	Oli
*L. helladae*	S	X	D, DA	Y	Y	Y	He	S-C, C-A	AP	Oli
*L. nanningensis*	D	A	DA, B, ScP	Y	Y	Y	Ho	C	AP, RN	Oli
*Q. intermedium*	D,S	O, A	D, DA	Y	Y	Y	He	C	AP	Mio
**Modern**										
*T. doichagensis*	D	A	DA, B	Y	Y	N	He, Ho	C	AP	
*T. verticillata*	D	?	D, DA, B	Y	Y	N	He	C	AP	
*T. excelsa*	D	A	D, DA, B	Y	Y	N	He	C	AP	
*L. edulis*	D	A	DA, B	Y	Y	Y	He	C	N	

The table summarizes the most important anatomical characters relevant for distinguishing fossil and extant Fagaceae woods. Abbreviations: Por = porosity: D = diffuse–porous; S = semi-ring porous. IVP = intervessel pitting: A = alternate; O = opposite; X = not observed; ? = uncertain. AP = axial parenchyma: D = diffuse; DA = diffuse-in-aggregates; B = banded; ScP = scanty paratracheal. Ex1s = exclusively uniseriate rays (Y = yes). Agg = aggregate rays (Y = yes). Ray2 = rays of two distinct size classes (Y = yes). RayCmp = ray composition: He = heterocellular; Ho = homocellular. RT = ray type: S-C = semi-compound; C-A = compound-aggregate. Cry = crystals: AP = in axial parenchyma; RN = in ray parenchyma; N = none. Age = stratigraphic age of fossil specimens (Oli = Oligocene; Mio = Miocene).

***Lithocarpoxylon nanningensis*** Huang, Jin, Quan et Oskolski sp. nov. (Huang *et al*., 2018), from the upper Oligocene of Guangxi, South China, differs from our specimen by its markedly denser vessel distribution and a more pronounced radial vessel arrangement, occasionally forming diagonal or flame-like patterns. It is further characterized by predominantly uniseriate, homocellular rays, only rarely biseriate, and by the presence of prismatic crystals chambered within axial parenchyma cells. These rays consist mainly of procumbent cells, with upright or square marginal cells occurring only sporadically. In contrast, the Přerov specimen exhibits a more complex ray architecture with well-developed semi-compound rays, typically 2–6 cells wide and often forming aggregated structures. The rays are consistently heterocellular, with clearly differentiated marginal upright or square cells, indicating a higher degree of stratification. Moreover, the Přerov material displays distinctly palisade-like vessel-ray pits with reduced borders – an anatomical feature not reported in *L. nanningensis* – further supporting their distinction.

***Quercoxylon intermedium*** Petrescu & Velitzelos from the Oligocene of Rhodopes, Bulgaria (Iamandei *et al*., 2012, 2016), and from the Late Miocene of Moldavia (Iamandei *et al*., 2022) shares anatomical feature such as chambered parenchyma containing crystals. However, it differs markedly in having a semi-ring porous wood structure with a distinct transition from large earlywood vessels to small latewood vessels arranged in radial bundles – features typical of deciduous white oaks. In contrast, our specimen exhibits a diffuse–porous vessel distribution, with no distinct growth ring boundaries. Moreover, the rays in *Q. intermedium* are frequently dissected by libriform fibres and form compound–aggregate structures, while the Přerov wood possesses narrower, undivided semi-compound rays that are aggregated but structurally continuous. This combination of diffuse porosity, undissected semi-compound rays and palisade-like vessel-ray pits distinguishes the Přerov material from *Q. intermedium* and supports its closer affinity to evergreen *Quercoxylon* species.

***Quercoxylon* (*Lithocarpoxylon*) *helladae*** is a fossil wood species attributed to evergreen members of Fagaceae. The type material consists of silicified trunks preserved in volcaniclastic deposits of Lesbos, Kastoria and Thrace (northeastern Greece), dated to the middle–late Oligocene (ca. 28–23 Ma).

To assess the taxonomic affinity of the Přerov fossil wood, we compared it with *Quercoxylon* (*Lithocarpoxylon*) *helladae* from Selmeier’s reference collection. The type material from Thrace exhibits indistinct growth-rings, semi-ring porosity, solitary vessels in dendritic to flame-like arrangements, vasicentric tracheids and chambered axial parenchyma with prismatic crystals. Particularly diagnostic is the ray system, where multiseriate rays that are compound-aggregate alternate with semi-compound rays arranged in vertical aggregates.

A primary diagnostic difference concerns wood porosity and growth-ring expression: Pr_17 shows a diffuse-porous structure with indistinct growth boundaries, whereas the Greek type material exhibits semi-ring-porous wood with well-defined boundaries.

**Fig. 4.**
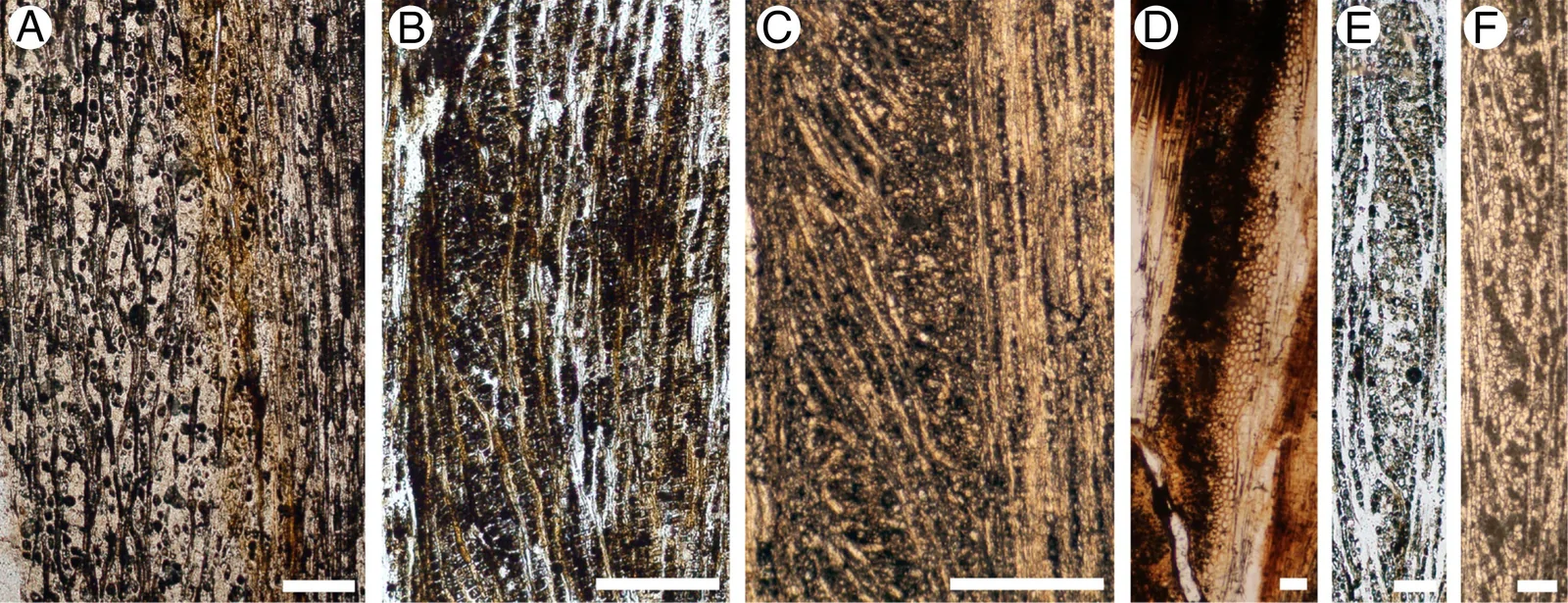
Tangential sections of fossil wood assigned to *Quercoxylon* aff. *helladae* (A–F). (A–C) Semi-compound rays: Czech Republic (A) Přerov County (Pr_17); (B) Žabčice (Zab_183). Greece (C) Selmeier Collection, Thrace. (D–F) Compound–aggregate rays: Czech Republic (D) Přerov County (Pr_42); (E) Žabčice (Zab_183). Greece (F) Selmeier Collection, Thrace. Scale bars: 100 µm (A–C); 50 µm (D–F). Classification and comparison of ray types are based on vessel-ray types after Petrescu (1976), Noshiro and Sasaki (2011) and Wheeler *et al*. (2022), as applied in the table of Mantzouka *et al*. (2021).

Ray variation is present among the Czech samples: Pr_42 and Zab_183 develop compound–aggregate rays dissected by libriform fibres, locally reaching 8–14 cells in width, while semi-compound rays in Pr_42 are only weakly developed, similar to the Greek material. In contrast, Pr_17 exhibits only semi-compound rays, 2–6 cells wide, locally aggregated but not dissected by libriform fibres. The Greek material more frequently displays compound–aggregate rays dissected by libriform fibres, locally up to 16 cells wide, with semi-compound rays remaining only weakly developed (Fig. 4).

The absence of compound–aggregate rays dissected by libriform fibres in sample Pr_17 is probably attributable to taphonomic loss or intrastem variation rather than any taxonomic difference (Schweingruber *et al.*, 2011). Overall, these ray characteristics correspond to those observed in evergreen oak lineages (Aloni, 1991; Suzuki and Ohba, 1991; Carlquist, 2001; Kobayashi *et al*, 2019; Gupta and Gupta, 2020; Wheeler *et al*., 2022).

In comparison with the Greek reference material (Fig. 4), all four specimens exhibit a similar ray system dominated by heterogeneous cell composition, with uniseriate to biseriate rays that locally merge into semi-compound or aggregated forms. Broad multiseriate rays in Pr_42 and Zab_183 match those in the Greek sample, while they are absent in Pr_17. Collectively, these observations support assignment of the Czech material to *Quercoxylon* aff. *helladae*.

*Comparison with modern woods*

Specimen Pr_17 from Želatovice displays solitary, rounded vessels, a dendritic vessel arrangement and aggregate rays – features that are diagnostic of the family Fagaceae. Additional anatomical traits, including indistinct growth rings, vasicentric tracheids, vessel-ray pits with reduced borders (palisade type) and predominantly uniseriate rays, correspond well to wood anatomy of evergreen Fagaceae genera such as *Quercus*, *Lithocarpus* and *Castanopsis* (Suzuki and Ohba, 1991).

A comparative search using the InsideWood database (2004–present), based on a combination of features (diffuse–porous wood, simple perforation plates, alternate intervessel pits, absence of helical thickenings, presence of vasicentric tracheids, axial parenchyma and aggregate rays), yielded the closest matches with *T. excelsa*, *T. verticillata* and *T. doichagensis* (A.Camus) Nixon & Crepet, and *Lithocarpus edulis* (Makino) Nakai.

Among these taxa, ***Trigonobalanus* (*Colombobalanus*) *excelsa*** differs from the fossil wood mainly in having slightly larger tangential vessel diameters (100–200 vs. 66–190 µm) and a more consistently expressed heterogeneous ray structure. Axial parenchyma in the fossil specimen also includes locally scanty paratracheal forms not regularly observed in *T. excelsa*. These differences are relatively minor and mostly quantitative.

***Trigonobalanus verticillata*** differs by smaller mean vessel diameters (50–100 µm), an apparent absence of crystals in ray cells and weaker evidence for stratified ray structure.

***Trigonobalanus* (*Formanodendron*) *doichangensis*** differs by more frequent homocellular ray sectors and a lower degree of ray stratification; it also shows significantly smaller tangential vessel diameters (Cutler, 1964; Itoh *et al*., 2022) and lacks well-developed septate/chambered axial parenchyma.

***Lithocarpoxylon edulis***, in contrast, more commonly exhibits homocellular rays with limited marginal cell differentiation, lacks prismatic crystals in ray tissue, and displays a more uniform, less complex ray architecture – yielding a poorer anatomical match to the fossil material.

*Growth ring comparison:* Fossil woods of the evergreen-type Fagaceae assigned to the genera *Lithocarpoxylon* and *Quercoxylon* are characterized predominantly by indistinct to absent growth rings, as documented in Oligocene occurrences of *Quercoxylon helladae* (Selmeier and Velitzelos, 2000) and *Q. intermedium* (Iamandei *et al*., 2012, 2016) as well as in *Lithocarpoxylon nanningensis* (Huang *et al*., 2018). In contrast, younger fossil material of *Q. intermedium* of Miocene age (Iamandei *et al*., 2022) already exhibits distinctly developed growth rings, showing a contrast between older and younger fossil material. Comparison with extant evergreen Fagaceae, particularly the genus *Trigonobalanus*, indicates a similar pattern dominated by absent to weakly expressed growth rings, while clearly developed seasonal growth zonation remains uncommon (Cutler, 1964; Mennega, 1980; Itoh *et al*., 2022).

Although intraspecific variability is well documented within extant Fagaceae, the combined evidence from vessel dimensions, ray heterogeneity and the presence of prismatic crystals in chambered axial parenchyma indicates that Pr_17 most closely corresponds to *Trigonobalanus excelsa* among the compared living taxa.

Fossil woods resembling *Trigonobalanus* have been reported from various Palaeogene and Neogene sites in Europe and Asia, suggesting a formerly broader range. Today, the lineage persists as a few relict species in subtropical and tropical montane forests of South America and Southeast Asia (*T. excelsa*, *T. doichagensis*, *T. verticillata*) (Manos *et al.*, 2008).

In conclusion, the Přerov fossil wood shows the closest anatomical affinity to evergreen Fagaceae and most closely resembles *Trigonobalanus* spp., particularly *T. excelsa*. This affinity is consistent with a warm-temperate to subtropical environment in the Western Carpathians during the Oligocene. However, due to the limited fossil record and possible anatomical convergence within evergreen Fagaceae, the assignment remains tentative at the genus level.

### Fossil coprolites

**Fig. 5.**
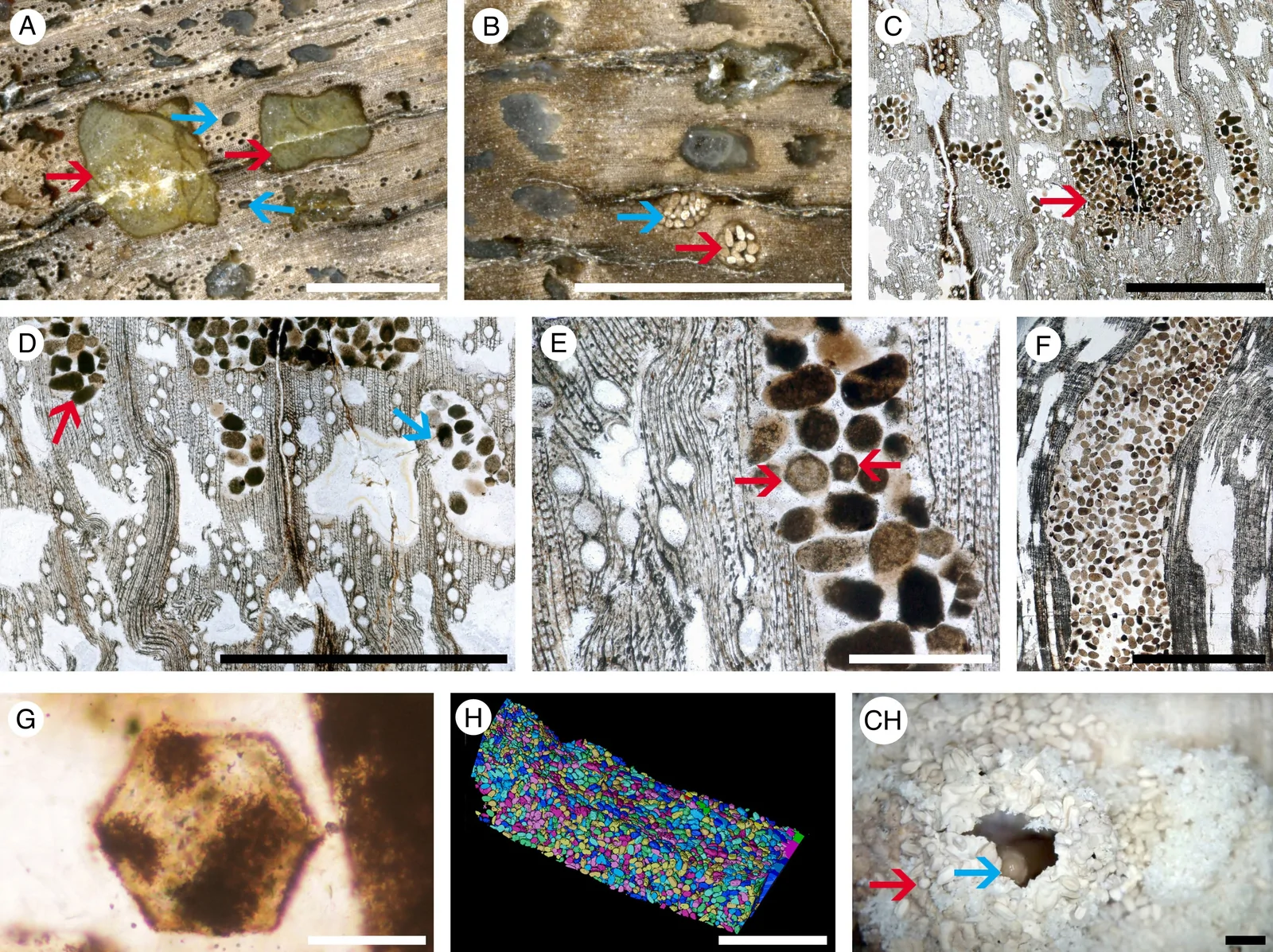
Pr_17. Kalotermite coprolites. (A–B) Transverse polished sections: star-like (arrow) and rounded (blue arrow) galleries, some containing coprolites. (C–E) Transverse thin sections: galleries filled with coprolites (arrows), with hyphae extending from pellets (arrow) into adjacent wood; distinct hexagonal forms visible. (F–G) Radial thin sections: star-like galleries occluded by coprolites and detail of a hexagonal pellet. (H) 3D micro-CT reconstruction of a coprolite-filled gallery showing dense packing consistent with termite activity. (CH) Modern analogue: *Kalotermes flavicollis* colony sealing a hole with excrement (arrow) and producing a slightly hexagonal pellet (arrow) (after Tretyakov, 2020). Scale bars: A–D, F, H–CH = 5 mm; E = 1 mm; G = 100 μm.

The distinctive hexagonal cross-sectional outline of the coprolites (Fig. 5), visible in cross-section, provides a key diagnostic feature for identifying their biological origin. This geometry is characteristic of faecal pellets produced by termites of the basal family Kalotermitidae, as documented in numerous studies (Light, 1929; Rogers, 1938; Knobloch, 1971; Rohr *et al*., 1986; Boucot, 1990; Rozefelds and Baar, 1991; Cabrera and Scheffrahn, 2001; Brammer and Scheffrahn, 2002; Sutherland, 2003; Francis and Harland, 2006; Pires and Sommer, 2009; Colin *et al*., 2011; Massini and Pujana, 2013; Jud *et al*., 2017; Bobadilla *et al*., 2020; Knaus, 2020; Moreau *et al*., 2020; Zega *et al*., 2020; Greppi *et al*., 2022; Dong *et al*., 2022; McLoughlin *et al*., 2024).

The hexagonal cross-section of the coprolites reflects the action of six rectal pads lining the termite hindgut, which compress and dewater the faecal material during pellet formation. The resulting geometry is therefore best interpreted as a mechanical consequence of this compression process, while the associated water reabsorption represents a key physiological adaptation in dry-wood habitats (Sutherland, 2003; Moreau *et al*., 2020).

*Type ichnospecies: Microcarpolithes hexagonalis*  Vangerow, 1954

*Description*

The galleries within the degraded wood (Pr_17, Pr_42) form a network of interconnected corridors that create vertically oriented cavities aligned with the trunk axis, with occasional radial expansions. Their arrangement in relatively regular rows suggests a correspondence with tentative growth-ring boundaries. Two main gallery types can be distinguished:

1. star–shaped in cross-section and spindle-like in radial section varying in dimensions from 60 × 50 to 20 × 20 mm; and
2. rounded to elongate with dimensions of about 0.3 × 1 mm.

In polished cross-sections, the galleries are bordered by degraded and corrugated vessel rays. While rarely interconnected, they are frequently filled with coprolites and frass embedded in a translucent quartz–chalcedony matrix. Tyloses in vessel lumina are occasionally removed by insect or fungal decay, resulting in partially collapsed vessel structures.

Coprolites are present within galleries and also in vessel lumen. They are not dispersed but rather densely accumulated in direct contact with one another and show random orientation. They are small, light brown, cylindrical to slightly prismatic, hexagonal in cross-section, sometimes with slightly rounded edges, and ovoid in radial section view. Their sides are relatively flat, though occasionally slightly concave. Internally, the coprolites are largely homogeneous, consisting predominantly of organic material with only minor inclusions of fragmented wood debris. In some cases, they also contain scattered, rounded conidia-like bodies and occasional remnants of fungal hyphae.

Coprolite sizes range from 167–357 µm (*n* = 67, mean = 270 µm, s.d. = 41 µm) in cross-section to 359–673 µm in radial section (*n* = 58, mean = 491 µm, s.d. = 79 µm). Volume ranges from 1.6 × 10^6^ to 7.3 × 10^6^ µm^3^ (*n* = 22, mean = 3.2 × 10^6^ µm^3^, s.d. = 0.8 × 10^6^ µm^3^), and the surface area ranges from 2.0 × 10^5^ to 9.3 × 10^5^ µm^2^ (*n* = 22, mean = 5.1 × 10^5^ µm^2^, s.d. = 0.9 × 10^5^ µm^2^). The sizes of conidia-like bodies in coprolites range from 6 to 17 µm (*n* = 14, mean = 10 µm, s.d. = 3 µm). The quantitative parameters of the coprolites are summarized in Table 3.

**Table 3.** Quantitative parameters of coprolites.

Structure	Size range	Mean	s.d.	*n*
Coprolite diameter (cross-section) (µm)	167–357	270	41	67
Coprolite diameter (radial-section) (µm)	359–673	491	79	58
Coprolite volume (µm^3^)	1.6 × 10^6^–7.3 × 10^6^	3.2 × 10^6^	0.8 × 10^6^	22
Coprolite surface area (µm^2^)	2.0 × 10^5^–9.3 × 10^5^	5.1 × 10^5^	0.9 × 10^5^	22
Conidia-like bodies in coprolites (µm)	6–17	10	3	14

*Ichnotaxonomic affinities of coprolites.* Following the differential diagnostic criteria of Colin *et al*. (2011), McLoughlin *et al*. (2024) and Knaus (2020), *Microcarpolithes hexagonalis* is characterized by cylindrical to rod-shaped, columnar to rounded coprolites with a generally homogeneous internal composition and a distinctive hexagonal to subhexagonal cross-section indicative of a termite origin.

This morphology distinguishes them from superficially similar coprolites of curculionid larvae, which may produce subhexagonal forms but are typically irregular, lack a consistently developed hexagonal geometry, include rough frass and do not form discrete, well-organized pellets comparable to those produced by termites. Based on our own observations of faecal pellets produced by larvae of various groups of extant beetles (e.g. Curculionidae, Buprestidae, Cerambycidae, Ptinidae) we found no meaningful morphological correspondence with the studied material. Therefore, termites are considered the most plausible producers.

The coprolites described here conform to the diagnostic criteria of *M. hexagonalis* in both overall morphology and cross-sectional geometry. Their morphology and internal structure are consistent with those produced by kalotermitid termites and differ from coprolites attributed to mastotermitids, which are typically less regular, lack a well-defined hexagonal cross-section and exhibit greater internal heterogeneity. Although both Kalotermitidae and Mastotermitidae may produce hexagonal faecal pellets, the studied material more closely resembles kalotermitid pellets in its regular geometry and relatively homogeneous internal composition. The consistent morphology, relatively uniform size and occurrence as dense accumulations within gallery systems further support a termite origin and distinguish these coprolites from superficially similar structures produced by other arthropods.

The diagnostic features summarized in Table 4 are based on comparisons with both fossil and extant material described in previous studies (e.g. Rohr *et al*., 1986; Boucot, 1990;; Francis and Harland, 2006; Colin *et al*., 2011; Dong *et al*., 2022; McLoughlin *et al*., 2024), which provide a framework for distinguishing termite-derived pellets from morphologically similar coprolites of other arthropods.

**Table 4.** Diagnostic comparison of coprolite features in Kalotermitidae and Mastotermitidae.

Feature	Kalotermitidae	Mastotermitidae
Size	Highly variable	Generally larger
Shape	Regular hexagonal to sub-hexagonal (cylindrical pellets)	Sub-hexagonal to less regular, more variable
Internal structure	Generally homogeneous	More heterogeneous
Occurrence	Dense accumulations within galleries	Less organized accumulation

A comparison with published data (Table 5) shows that coprolite dimensions attributed to *M. hexagonalis* span a wide range across different stratigraphic intervals including recent *Kalotermes* faecal pellets and geographical regions. The studied specimens fall within this variability, toward the lower to intermediate size range, and overlap with values presented in Table 5. This variation is not considered taxonomically significant, as size alone is not a primary criterion for differentiating ichnotaxa (Bertling *et al*., 2022) and may instead reflect differences in producer size, ontogenetic stage or potentially taxonomic variability among producers, as suggested by the diversity of extant kalotermitid taxa.

**Table 5.** Comparison of published and studied coprolite dimensions of *Microcarpolithes hexagonalis*.

Authors	Year	Geological age	Locality	Dimensions (mm)
This study		Oligocene	Czechia	0.17–0.36 (0.27) × 0.36–0.67 (0.49)
**Minimum and maximum values (published studies)**				0.26–3.21 × 0.22–1.16
Dong *et al*. (2022)		Aptian	Inner Mongolia	0.67–3.21 (1.94) × 0.25–1.16 (0.7)
Greppi *et al*. (2022)		Albian	Argentina	0.52–1.17 (0.8) × 0.41–0.77 (0.59)
Francis and Harland (2006)		Barremian	UK	0.4–0.6 (0.5) × 0.4–0.5 (0.45)
Colin *et al*. (2011)		Late Albian	France	0.26–1.45 (0.85) × 0.30–0.40 (0.35)
McLoughlin *et al*. (2024)		Albian–Cenomanian	Australia	0.97–1.62 (1.29) × 0.59–1.0 (0.79)
Jud *et al*. (2017)		Coniacian	Canada	1.13 × 0.73
Heřmanová *et al*. (2021)		Cenomanian	Czechia	0.7 × 1–0.5
Rohr *et al*. (1986)		Upper Cretaceous	USA	0.75 × 0.5
Moreau *et al*. (2020)		Priabonian–Rupelian	France	0.47–0.62 (0.54) × 0.22–0.32 (0.27)
Knobloch (1971)		Palaeogene	Czechia	0.7–1.75 (1.2) × 0.45–0.94 (0.6)
Rozefeld and DeBaar (1991)		Rupelian	Australia	2.0–2.6 (2.3) × 0.8–1.0 (0.9)
Massini and Pujana (2013)		Miocene	Argentina	0.91 × 0.45
Sutherland (2003)		Aquitanian–Burdigalian	New Zealand	1.0 × 0.5
Rogers (1938)		Neogene (Pliocene)	USA	0.7–0.9 (0.8) × 0.3–0.5 (0.4)
Colin *et al*. (2011)		Recent (Holocene)	Africa	0.64 × 0.30

Dimensions are reported as length × width. Values in parentheses represent calculated mean values, while the accompanying values indicate the ranges reported by the original authors.

### Fungal remains

Both fossil wood specimens (Pr_17, Pr_42) exhibit clear evidence of white rot, specifically of the pocket-rot type, indicated by characteristic macroscopic pocket-like cavities (Černý, 1989). Microscopic observations show that fungal degradation predominantly affected the secondary xylem, with hyphae extensively penetrating the tissue. The decay pattern corresponds to selective delignification, in which lignin is removed while cellulose-rich cell walls remain largely intact (Blanchette, 1982; Otjen and Blanchette, 1984; Blanchette, 1984). Such a mode of decay is typical of basidiomycetes responsible for wood degradation in oxygenated environments.

### Microscopic description

Fungal hyphae form irregular filamentous networks throughout the fossil wood matrix, commonly occupying cell lumina, vessel-ray parenchyma, vasicentric tracheids, galleries and decay pockets. The hyphae are tubular, regularly septate and frequently branch into dense mycelial structures, with secondary filaments narrower than the parent hyphae. Both simple septa and clamp connections are present, and in some cases the proliferation of new hyphae through clamp connections was observed.

**Fig. 6.**
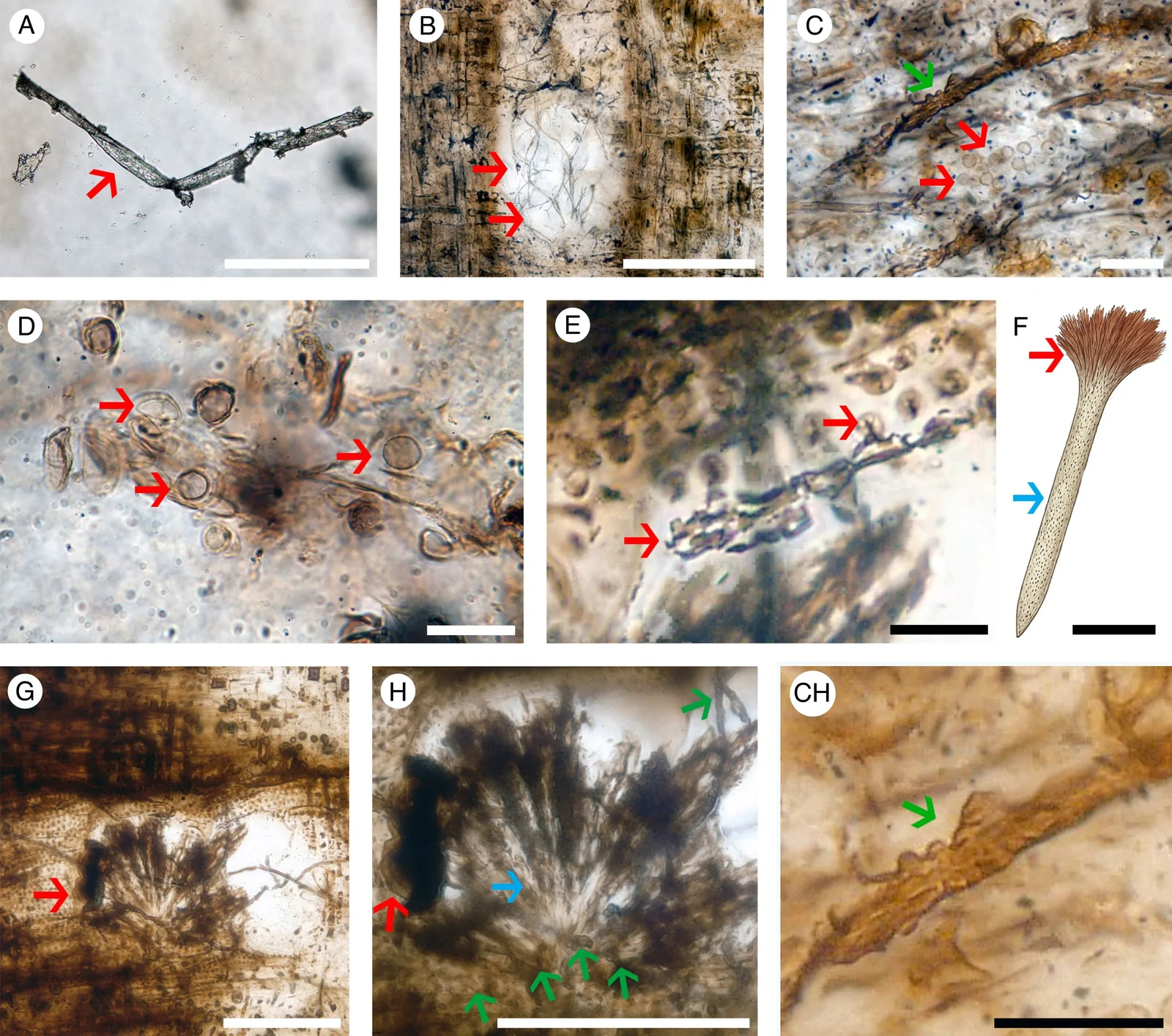
Pr_42. Fungal structures associated with termite galleries. (A–B) Hyphae embedded within coprolite-filled galleries and dense mycelial networks in delignified pockets. (C–E) Clamp connection (arrow), spores (arrows) and (D) conidia (arrows) associated with hyphae within a delignified pocket of the wood, and (E) dense mycelial network and hyphae proliferating into wood punctures. (F) Schematic reconstruction of fungal structures of uncertain affinity with an expanded head (arrow) and stalk (arrow). (G–H) Structures of uncertain affinity in thin section, consisting of a stipe (arrows) and a tufted capitulum (arrows), arising from the hyphal network (arrows). (CH) Detail of a clamp connection (arrow). Scale bars: A, B, G–H = 200 μm; C–F, CH = 20 μm.

The regions surrounding vessels and vessel-ray cells are particularly degraded, with poorly preserved palisade pits and widespread disruption of the secondary xylem. Numerous rounded fungal structures, interpreted as conidia, occur in association with pigmented hyphal clusters. These bodies are thin-walled, ellipsoid to spherical, and contain distinctly demarcated nuclei surrounded by a pigmented rim, indicating asexual reproduction typical of anamorphic fungi (Fig. 6D). These may represent asexual propagules or residual products of fungal decay.

A septate, fusiform hypha with thin to moderately thick walls and occasional lateral outgrowths was also documented, probably representing an actively growing generative hypha. In one degraded region, a tangle of thick-walled, pigmented hyphae occurred together with a cluster of fungal spores (2–5 µm in diameter), indicating advanced stages of decay (Fig. 6C).

From some of these hyphal networks, bristle-like tufts (Fig. 6F–H) emerge, interpreted as putative fungal structures of uncertain affinity, commonly occurring adjacent to vessel walls and surrounded by vasicentric tracheids. These structures exhibit a bipartite organization, consisting of a basal stipe (approximately two-thirds of the total length) and a terminal portion (one-third). The basal part represents a localized concentration of hyphae of varying diameter that extend into the surrounding wood tissue, whereas the terminal portion appears as a bristle-like structure composed of fine filamentous elements. These filaments locally display reddish to brownish coloration, which may reflect original biological pigments (e.g. melanin or phenolic compounds) or diagenetic staining by iron oxides. The morphology of these structures has no clear analogue in the available literature; however, their occurrence within hyphal aggregates suggests a fungal origin, although their precise affinity cannot be determined with confidence (Fig. 6).

The width of hyphae ranges from 4 to 13 µm (*n* = 16, mean = 9 µm, s.d. = 2 µm). Conidia-like bodies range in diameter from 9 to 32 µm (*n* = 26, mean = 18 µm, s.d. = 5 µm). The central nucleus within the conidia ranges from 7 to 22 µm (*n* = 25, mean = 12 µm, s.d. = 4 µm), while the outer rim measures 4–6 µm (*n* = 25, mean = 4 µm, s.d. = 0.9 µm). Fungal spores, observed in clusters within hyphal networks, measure between 2 and 5 µm in diameter (*n* = 17, mean = 3.6 µm, s.d. = 0.8 µm), indicating a smaller reproductive stage distinct from conidia. The total size of fungal structures of uncertain affinity ranges from 136 to 299 µm (*n* = 15; mean = 221 µm; s.d. = 51 µm). The quantitative parameters of the fungal structures are summarized in Table 6.

**Table 6.** Quantitative parameters of fungus.

Structure	Size range (µm)	Mean (µm)	s.d. (µm)	*n*
Width of hyphae	4–13	9	2	16
Diameter of conidia-like bodies	9–32	18	5	26
Diameter of conidial nucleus	7–22	12	4	25
Conidial outer rim	4–6	4	0.9	25
Diameter of fungal spores	2–5	3.6	0.8	17
Structures of uncertain affinity	136–299	221	51	15

*Possible affinities of the fungus.* The occurrence of branched mycelium invading vessel lumina, ray cells and tracheids (Fig. 6E), together with septate hyphae and clamp connections, provides reliable anatomical evidence for fungal infection. These features are characteristic of extant basidiomycetes, particularly those responsible for white pocket rot, which preferentially colonize vessels and ray tissues (Käärik, 1974). Among the most consistently reported attributes of this fungal group is the presence of hyphal clamp connections, documented across numerous studies (Stubblefield *et al*., 1985; Stubblefield and Taylor, 1986; Lindquist and Isaac, 1991; Srivastava *et al*., 2009; Feng *et al*., 2015; Taylor *et al*., 2015; Wan *et al*., 2016*a*, *b*; Biswas *et al*., 2020; Tian *et al*., 2020; Xie *et al*., 2023).

The presence of clamp connections (Fig. 6C, H) indicates that the fungus can be assigned to the Basidiomycota. Within the wood structure, bristle-like tufts attached to cell lumina were observed, interpreted as structures of uncertain affinity, tentatively attributed to a possible fungal origin. Spores were also observed within the mycelium; however, their preservation does not permit evaluation of diagnostic characters. Consequently, the systematic affinities of the fungus cannot be resolved with confidence beyond the basidiomycete level.

## DISCUSSION

The fossil wood specimens studied here exhibit diagnostic anatomical features consistent with *Quercoxylon helladae*, including diffuse–porous wood, indistinct growth rings, solitary vessels and semi-compound rays – traits characteristic of evergreen thermophilous oaks within Fagaceae. These features closely match those observed in material from Oligocene sites in the Thrace region of Greece, where *Q. helladae* co-occurs with leaf imprints of *Eotrigonobalanus furcinervis* (Velitzelos *et al*., 1999; Velitzelos *et al.*, 2014; Selmeier and Velitzelos, 2000). The Přerov material likewise shares these diagnostic traits, supporting a comparable palaeoenvironmental origin. This taxonomic and ecological association is reinforced by the co-occurrence of *E. furcinervis* and *Quercoxylon*-type wood in redeposited Menilite facies at several sites across the Western Carpathians, including the Šitbořice Beds.

A key feature in this context is the indistinct to absent growth ring structure, which is also observed in the Přerov material. This phenomenon warrants ecological interpretation beyond anatomical description.

### Synthesis of taxonomic, taphonomic and stratigraphic evidence

Anatomical features of *Quercoxylon helladae*, the morphology of hexagonal coprolites, associated fungal structures, stratigraphic parallels (e.g. Pavlovice, Krumvíř, Žabčice) and the co-occurrence with *Eotrigonobalanus furcinervis* collectively indicate that the Přerov fossil wood was redeposited from Oligocene Menilite facies rather than formed *in situ* within Badenian tuffs (Továrková, 2009, 2011) or Quaternary alluvium. Together with the Žabčice specimen, these finds fit into a broader regional pattern of Menilite-derived fossil wood redeposited into younger sediments, underscoring the role of the Menilite facies as a primary source of exceptionally silicified plant remains that preserve biotic interactions otherwise rarely recorded in the fossil record.

### Taphonomic sequence of fossil wood

Because the taphonomic processes of silicified woods redeposited from the Menilite Formation remain insufficiently understood, we propose a plausible reconstruction based on anatomical evidence, fungal–termite interactions and sedimentological context. The initial stages of this sequence, including colonization of the wood by clamp-bearing basidiomycete hyphae and subsequent termite activity, are directly supported by anatomical observations (e.g. white pocket rot and coprolite-filled galleries). These features suggest that fungal degradation probably preconditioned the wood for colonization by drywood termites (Kalotermitidae), which subsequently utilized or locally expanded pre-existing decay zones.

The later stages of the taphonomic sequence, particularly those involving transport and redeposition, are more interpretative and are not directly supported by the available evidence. The studied material was recovered from Quaternary terraces; however, comparable silicified woods are also known directly from the Menilite Formation, which represents a marine depositional environment. The studied localities occur in close spatial proximity (on the order of a few kilometres) to this formation or are associated with its weathering cover (see Table 5). It is therefore likely that the wood underwent transport from its original terrestrial setting into the marine system, for example via fluvial input followed by transport within coastal or marine environments. The extent of transport cannot be determined directly; however, the consistent macroscopic and anatomical characteristics of the silicified woods, including their bituminous odour, coloration and chalcedony textures, indicate a common source within the Menilite Formation. Even in the case of transport, the original source area would thus remain within the Central European domain. The proposed sequence should therefore be understood as a combination of directly supported observations and plausible taphonomic interpretation.

### Termite activity and fungal co-occurrence in fossil wood

Several specimens contain hexagonal coprolites consistent with the faecal pellets of basal Kalotermitidae. In extant species such as *Kalotermes flavicollis*, nesting behaviour involves the accumulation of faecal material and wood debris within galleries (Roisin, 2000; Nalepa, 2015). While fungal symbioses are well documented in some termite groups (e.g. Macrotermitinae), such associations are not established for Kalotermitidae. The basidiomycete structures documented here are therefore more plausibly interpreted as environmental wood-decay fungi that either co-occurred with termite activity or were opportunistically exploited. Although the fossil record of termite–fungus associations is rare, comparable co-occurrences of coprolitic structures and fungal remains have been reported from fossil wood (Grimaldi and Engel, 2005; Genise, 2017). The Přerov specimens document a close spatial association between fungal structures and termite-produced coprolites; however, this association is interpreted cautiously and may reflect co-occurrence within the same substrate, potentially indicating opportunistic fungivory.

### Interpreting growth ring absence in palaeobotanical contexts

In both fossil and extant woods, growth rings may be absent or poorly defined, a feature typically associated with species growing under aseasonal climatic regimes (Worbes, 2002; Brienen and Zuidema, 2005; Sass-Klaassen *et al*., 2008). In tropical and subtropical environments with minimal variation in temperature and precipitation, cambial activity may proceed year-round, preventing the formation of distinct ring boundaries. Evergreen trees with slow or episodic growth likewise lack regular dormancy, further contributing to indistinct increments (Carlquist, 2001). Anatomically, this condition is expressed in diffuse-porous wood with irregular parenchyma distribution and relatively homogeneous vessel diameters, complicating ring boundary recognition (IAWA Committee, 1989). The indistinct nature of growth-ring boundaries is widely documented in tropical and subtropical taxa and reflects both ecological controls and anatomical properties of the wood, particularly diffuse-porous structure and overall homogeneity of the xylem, which limit the formation of distinct growth increments (Tarelkin *et al*., 2016). While taphonomic and pathological processes may locally reduce the visibility of growth-ring boundaries, this feature is not diagnostic on its own. However, in the present material, the consistent occurrence of indistinct or absent rings in otherwise well-preserved specimens is more plausibly interpreted as reflecting growth under stable, humid conditions with weak seasonality (Brienen and Zuidema, 2005; Tarelkin *et al*., 2016), rather than solely a taphonomic overprint.

By analogy with modern relatives, two scenarios are plausible for Oligocene forests with *Quercoxylon helladae*. One possibility is humid evergreen rainforests resembling present-day tropical and subtropical cloud forests of Southeast Asia, where *Trigonobalanus verticillata* (Malaysia, Indonesia, Philippines) and *T. doichangensis* (southern China, Vietnam, Thailand) grow under perhumid conditions. A third extant analogue – *Trigonobalanus excelsa* from the Andean cloud forests of Colombia – shows the closest anatomical affinity to the Přerov material and likewise occupies warm, extremely humid montane forests with low seasonality. Alternatively, these Oligocene forests may have formed part of mixed mesophytic assemblages with evergreen oaks and subtropical conifers, comparable to relict evergreen broad-leaved forests that persist today in southern China in mountainous regions with stable high humidity and dominance of *Lithocarpus*, *Quercus* sect. *Cyclobalanopsis* and related Fagaceae. All three analogues imply a perhumid climate with limited seasonality that supported complex saproxylic communities.

### CONCLUSIONS

Fossil wood assigned to *Quercoxylon helladae* from the Oligocene Menilite facies, together with associated saproxylic traces, provides a basis for reconstructing warm subtropical climate conditions in Central Europe during this interval. Evidence includes: (1) the occurrence of *Q. helladae*, taxonomically allied to *Trigonobalanus* that today grows exclusively in warm evergreen forests; (2) hexagonal termite coprolites, indicative of tropical and subtropical habitats; and (3) co-occurring leaves of *Eotrigonobalanus furcinervis*, an indicator of evergreen broad-leaved forests. Collectively, these lines of evidence offer valuable insight into the ecological dynamics and evolutionary history of saproxylic interactions in Oligocene ecosystems. Their close resemblance to Greek material – where *Q. helladae* co-occurs with *Eotrigonobalanus furcinervis* – supports a palaeobiogeographical link to warm-temperate to subtropical evergreen forests extending on a larger continental scale during the Oligocene.

Several converging lines of evidence reinforce this interpretation.

- Anatomical traits such as diffuse porosity, solitary vessels and semi-compound rays forming occasional short aggregates correspond to evergreen thermophilous oaks within Fagaceae.
- Hexagonal coprolites assignable to *Microcarpolithes hexagonalis* indicate the activity of drywood termites (Kalotermitidae). Their ecology, closely tied to decomposition of dry, well-aerated wood in warm climates, suggests similar conditions prevailed during fossilization.
- Microscopic fungal structures – including septate hyphae with clamp connections, selective delignification and associated spores – are consistent with white-rot Basidiomycota.
- The close spatial association of fungal structures with termite coprolites suggests possible fungivory or simple co-occurrence within the same substrate.
- Despite advanced biological degradation, cellular preservation is exceptional, pointing to early mineralization under low-oxygen, organic-rich conditions typical of Menilite facies.
- Silicification patterns, bituminous odour and regional comparisons (Žabčice, Pavlovice, Kelč, Krumvíř) reveal a consistent pattern of redeposition from Menilite strata across Moravia.
- Comparative anatomy with modern *Trigonobalanus excelsa* supports a palaeoecological reconstruction of moist subtropical forests with aseasonal growth conditions, reflected in the indistinct growth-ring boundaries of the fossil material.
- Micro-CT analysis provided novel 3D evidence of termite galleries and coprolite accumulations in fossil wood, further confirming ichnotaxonomic assignment and degradation pathways.
- The occurrence of *Microcarpolithes hexagonalis* in these Oligocene deposits represents a well-constrained record from Central Europe and contributes to refining the stratigraphic and palaeogeographical distribution of the ichnotaxon.
- The close resemblance of the Přerov specimens to Greek *Quercoxylon helladae* strengthens the palaeobiogeographical interpretation of a once broader distribution of evergreen fagaceous lineages, today restricted to relict populations of *Trigonobalanus* in Southeast Asia and South America.

This study provides the first direct evidence of a termite–fungus interaction in anatomically preserved Oligocene wood from Central Europe. It highlights the role of fungal decay in facilitating termite colonization and underscores the potential for mutualistic relationships in deep time.

Beyond its palaeoecological implications, the material demonstrates the exceptional archival value of permineralized wood for reconstructing biotic interactions, palaeoenvironmental conditions and diagenetic pathways in the Menilite palaeoecosystem.

Future work should aim to identify similar associations in other Menilite localities and across broader palaeogeographical settings, thereby refining the climatic and environmental framework of Oligocene Central Europe. Such evidence would help determine whether subtropical evergreen forests with saproxylic termite–fungus communities were regionally widespread or restricted to localized refugia within the Northern Hemisphere.

## Figures and Tables

**Fig. A1.**
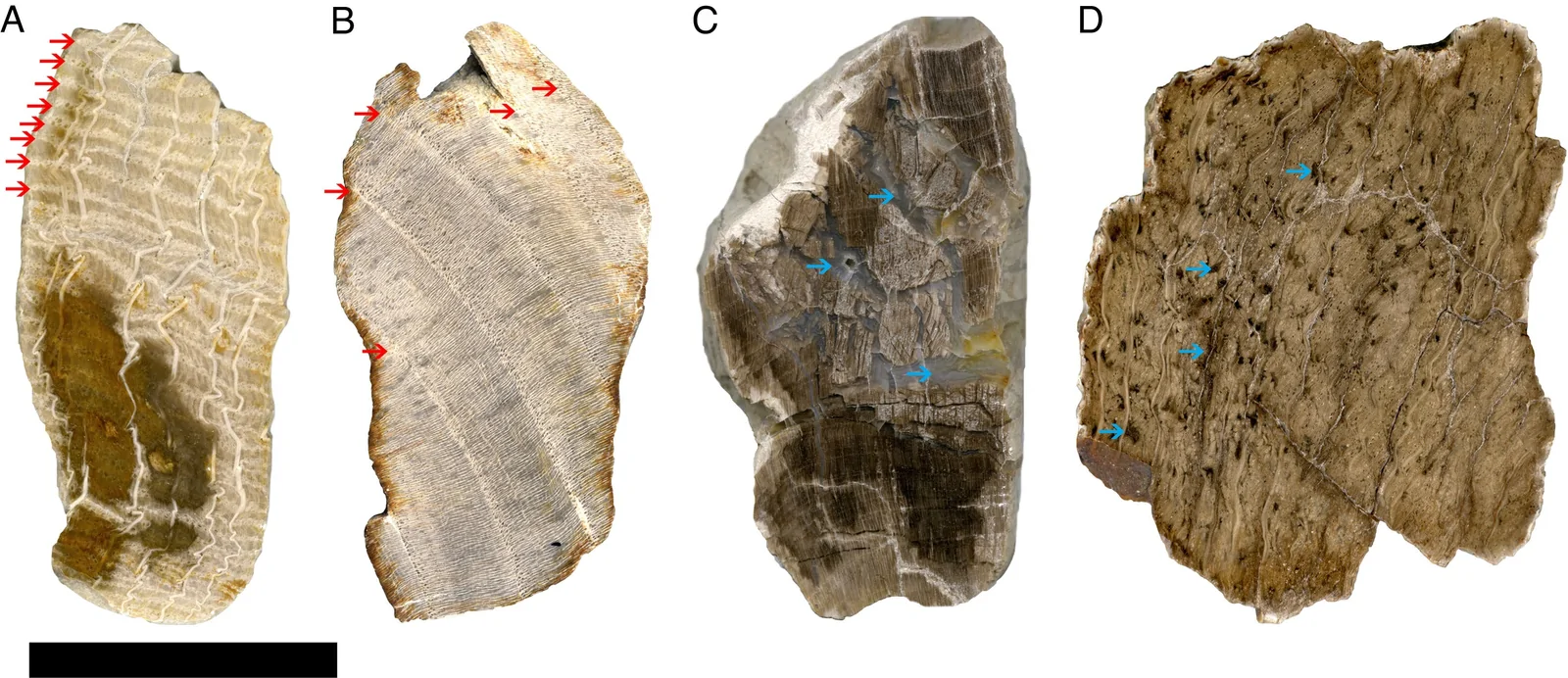
(A–B) Neogene (Miocene) specimens from Žabčice with a thick light patina, incomplete silicification without chalcedony and distinct growth rings: (A) Quercoxylon sp.; (B) Ulmoxylon sp. (C–D) Palaeogene (Oligocene) specimens from Přerov with a thin patina, chalcedony infilling and no distinct growth rings: (C) coniferous wood; (D) Quercoxylon aff. helladae.

**Fig. A2.**
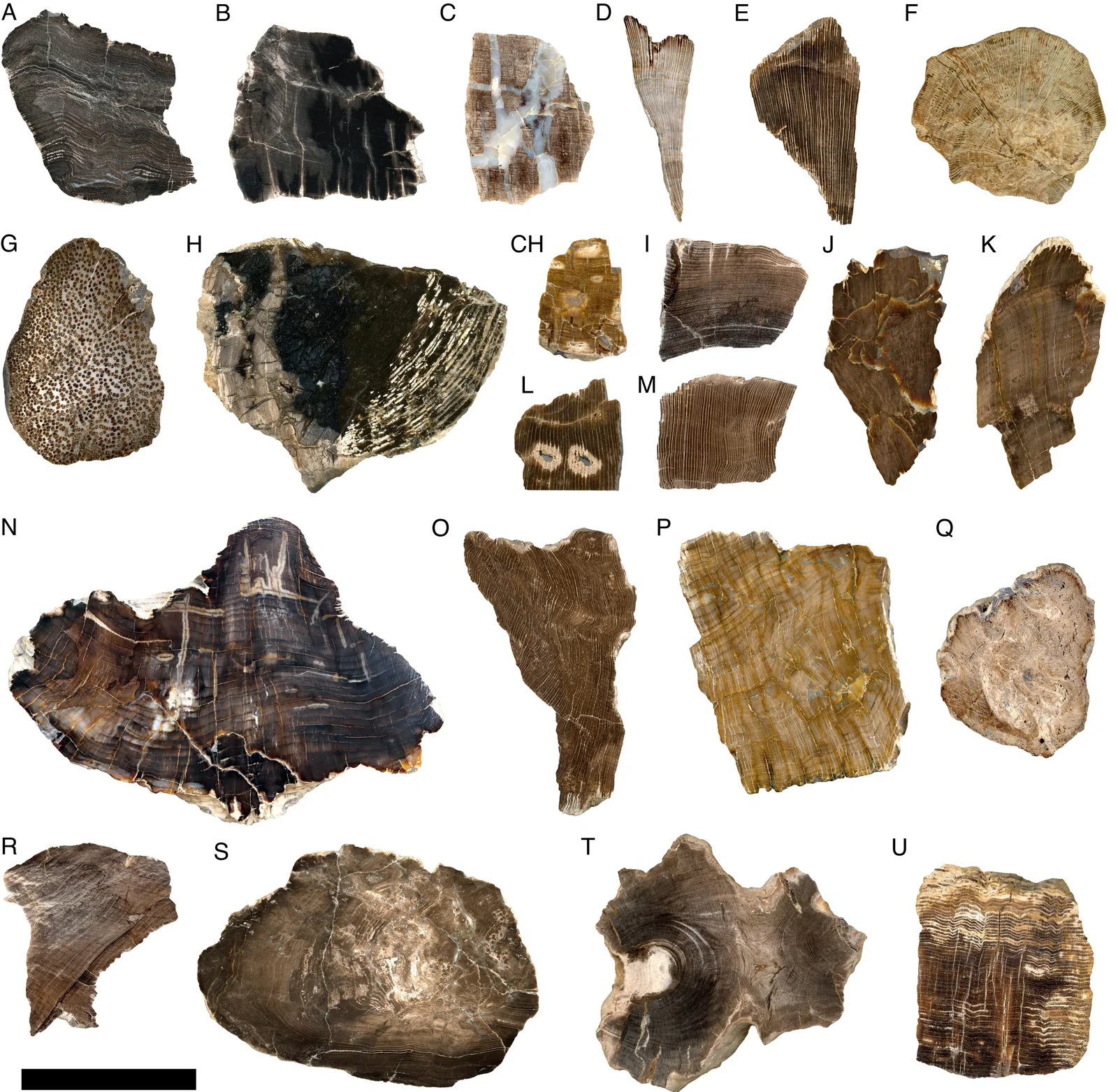
Fossil wood specimens arranged approximately from north to south Moravia. (A) Conifer, Hranice (Oldřich Krejčí, ČGS); (B) conifer, Kelč (Alois Kovařčík); (C) conifer, Soběchleby (Miroslav Bubík, ČGS); (D–F) *Platanus*-type wood, Přerov; (G) *Palmoxylon*, Přerov; (H) conifer within silicified concretion, Přerov (Martin Charamza); (CH) conifer, Přerov; (I) conifer, Mouchnice; (J) evergreen oak wood type, Kroměříž; (K) evergreen oak woodtype, Měnín (David Buriánek, ČGS); (L) *Spiroplatanoxylon*, Vážany nad Litavou (after Továrková *et al.* 2014); (M) *Platanus*-type wood, Mouchnice; (N) conifer, Lipník nad Bečvou (Antonín Přichystal); (O) *Platanus*-type wood, Měnín; (P) conifer, Měnín; (Q) conifer, Krumvíř; (R) conifer, Velké Němčice; (S) conifer, Mikulov (Dan Volf); (T) conifer, Jestřabice; (U) conifer, Nemotice. Scale bar = 5 cm.

## AI ASSISTANCE

The authors acknowledge the use of artificial intelligence-based language tools, which assisted in linguistic editing and minor stylistic improvements of the manuscript text.

## APPENDIX

### Appendix 3

**Table A1.** Overview of diagnostic characters in fossil and modern oak woods (Fagaceae) compared with Pr_17, Pr_42 and Zab_183.

	Por	VArr	VGrp	PP	IVP	VRP	HT	MVTD (µm)	VT	FP	AP	Ex1s	Agg	Ray2	RayCmp	Cry	Age
**Fossil wood**																	
Pr_17	D	R, Dg	ExSol	Si	A	R	N	66–190	Y	D	Da, B, ScP	Y	Y	N?	He	Ap	Oli
Pr_42	D	R, Dg	ExSol	Si	A	R	N	75–180	Y	D	Da, B, ScP	Y	Y	Y	He	Ap	Oli
Zab_183	D	R, Dg	ExSol	Si	A	R	N	90–200	Y	D	Da, B, ScP	Y	Y	Y	He	Ap	Oli
*Q. helladae*	S	R, Dg	ExSol	Si	X	R	N	112–220	Y	D	D, Da	Y	Y	Y	He	N	Oli
*L. microporosum* *	D	R	ExSol	Si	X	R	N	53–302	Y	I	D	Y	Y	Y	Ho	N	Pli
*L. nanningensis*	D	R	ExSol	Si	A	R	N	45–243	Y	I	Da, B, ScP	Y	Y	Y	Ho(He)	Ap,Rp	Upper Oli
*L. ashwilii*	S,D	R, Dg	ExSol	Si	X	R	N	208–215;160–260	Y	D	D, Da	Y	Y	Y	Ho(He)	Ap	Eo
*L. radioporosum*	D	R	ExSol	Si	X	R	N	80–150	Y	D	Da, B	Y	Y	Y	Ho	Ap,Rp	Eo
*Q. intermedium*	S,D	R	ExSol	Si	O, A	R	N	200–350;40–150	Y	D	D, Da	Y	Y	Y	He	Ap	Upper Mio
*C. nanningensis*	D	R, Dg	ExSol, RM, (4+)	Si, Sc	A	R	N	54–289;21–90	Y	D	Da, B, ScP	Y	Y	Y	Ho(He)	Ap	Upper Oli
*C. guangxiensis*	D	R	ExSol(RM)	Si	A	R	N	56–247	Y	D	Da, B, ScP	Y	Y	N	Ho(He)	Ap	Upper Oli
*C. bulgarica*	D(S)	Dd	ExSol	Si	O, A	X	N	50–130	Y	D	Da, ScP, Vsi	Y	Y	Y	He	N	Late Mio-Early Pli
*C. makinoi*	S	R	ExSol	Si	X	R	N	35–195;43–252	Y	I	Da	Y	N	N	Ho	Ap	Pli
*C. uchiuraensis*	R	R	ExSol	Si, Sc	A	R	N	120–260	Y	I	X	Y	N	N	Ho(He)	Ap	Oli,Early Mio
**Modern wood**																	
*T. doichagensis*	D	R, Dg	ExSol	Si	A	R	N	66–190	Y	D	Da, B	Y	Y	N	He(Ho)	Ap	
*T. verticillata*	D	R, Dg	ExSol	Si	?	X	N	50–100	Y	D	Da, B	Y	Y	N	He	N	
*T. excelsa*	D	R	ExSol	Si	A	R	N	100–200	Y	D	D, Da, B	Y	Y	N	He	Ap	
*L. edulis*	D	Dg, R	ExSol	Si	A	R	N	100–200	Y	D	Da, B	Y	Y	Y	Ho	N	
*L. kawakami*	D	Dg, R, D	ExSol	Si	A	R	N	100–200	Y	D	ScP, Da, B	Y	Y	Y	Ho	N	
*L. indica*	D	Dg, R	ExSol	Si	A	R	N	100–200;<200	Y	?	Da, B	Y	Y	N	Ho	Ap,Rp	

Abbreviations: Por – porosity type [D, diffuse–porous; S, semi-ring porous; R, ring–porous; D(S), transitional diffuse/semi-ring]; VArr – vessel arrangement (R, radial; Dg, diagonal; Dd, dendritic); VGrp – vessel grouping [ExSol, exclusively solitary vessels; RM, radial multiples; (4+), groups of more than four vessels]; PP – perforation plates (Si, simple); IVP – intervessel pits (A, alternate; O–A, opposite to alternate; Sc, scalariform); VRP – vessel-ray pits (R, reduced; N, normal); HT – helical thickenings (X, absent; N, not observed); MVTD – mean vessel-tube diameter (µm); VT – vessel tyloses (Y, present; I, indistinct or rare); FP – fibre pits (D, distinct; I, indistinct); AP – axial parenchyma (Da, diffuse apotracheal; B, banded; ScP, scanty paratracheal; Vsi, vasicentric); Ex1s – exudates or crystals in vessels (Y, yes; N, no); Agg – aggregate rays (Y, yes; N, no); Ray2 – presence of two ray size classes (uniseriate and multiseriate); RayCmp – ray composition [He, heterogeneous; Ho, homogeneous; Ho(He), homogeneous with some heterogeneous features; He(Ho), heterogeneous with homogeneous tendency]; Cry – crystals (Ap, prismatic; Rp, rhomboidal; N, none); Age – stratigraphic age (Oli, Oligocene; Upper Oli, Upper Oligocene; Eo, Eocene; Upper Mio, Upper Miocene; Pli, Pliocene; Late Mio–Early Pli, Late Miocene to Early Pliocene).

^*^ Cheng *et al.* (2018).
